# Phonological Ambiguity Detection Outside of Consciousness and Its Defensive Avoidance

**DOI:** 10.3389/fnhum.2019.00077

**Published:** 2019-04-05

**Authors:** Ariane Bazan, Ramesh Kushwaha, E. Samuel Winer, J. Michael Snodgrass, Linda A. W. Brakel, Howard Shevrin

**Affiliations:** Program of Research on Unconscious Processes, Ormond and Hazel Hunt Laboratory, Department of Psychiatry, University of Michigan, Ann Arbor, MI, United States

**Keywords:** phonology, subliminal, unconscious, N320, ambiguity, avoidance, consciousness, defense

## Abstract

Freud proposes that in unconscious processing, logical connections are also (heavily) based upon phonological similarities. Repressed concerns, for example, would also be expressed by way of phonologic ambiguity. In order to investigate a possible unconscious influence of phonological similarity, 31 participants were submitted to a tachistoscopic subliminal priming experiment, with prime and target presented at 1 ms. In the experimental condition, the prime and one of the 2 targets were phonological reversed forms of each other, though graphemically dissimilar (e.g., “nice” and “sign”); in the control condition the targets were pseudo-randomly attributed to primes to which they don't belong. The experimental task was to “blindly” pick the choice most similar to the prime. ERPs were measured with a focus on the N320, which is known to react selectively to phonological mismatch in supraliminal visual word presentations. The N320 amplitude-effects at the electrodes on the midline and at the left of the brain significantly predicted the participants' net behavioral choices more than half a second later, while their subjective experience is one of arbitrariness. Moreover, the social desirability score (SDS) significantly correlates with both the behavioral and the N320 brain responses of the participants. It is proposed that in participants with low SDS the phonological target induces an expected reduction of N320 and this increases their probability to pick this target. In contrast, high defensive participants have a perplexed brain reaction upon the phonological target, with a negatively peaking N320 as compared to control and this leads them to avoid this target more often. Social desirability, which is understood as reflecting defensiveness, might also manifest itself as a defense against the (energy-consuming) ambiguity of language. The specificity of this study is that all of this is happening totally out of awareness and at the level of very elementary linguistic distinctions.

## Introduction

While language is decoded consciously along semantic lines, different psychoanalytic authors, chiefly among whom Sigmund Freud, have stressed the importance of the word form, this is its phonology, when it comes to unconscious mental processes (for a systematic review, see Bazan, 2007, 2011). In the *Interpretation of dreams* Freud (1900/1958 p. 530; Italics added) proposes that “psychic elements” in free association are connected by associations, which are often based on “*assonance, verbal ambiguity*, and temporal coincidence, without inner relationship of meaning; in other words (…), they are connected by all those associations which we allow ourselves to exploit in *wit and playing upon words*.” Or again, for dreams: “The ideas which transfer their intensities to each other stand in the loosest mutual relations. They are linked by associations of a kind that is scorned by our normal thinking and relegated to the use of jokes. In particular, we find associations based on *homonyms and verbal similarities* treated as equal in value to the rest.” (Freud, 1900/1958, p. 596^1^). Repressed concerns, for example, would express themselves by way of phonologic ambiguity (Freud, 1901/1974). This is a pithy example: “a patient tried to attribute his nervousness to business worries (…) during the cotton crisis. He went on to say: ‘My trouble is all due to that d—frigid wave; there isn't even any seed to be obtained for new crops.' He referred to a cold wave which had destroyed the cotton crops, but instead of writing ‘wave' he wrote ‘wife.' In the bottom of his heart he entertained reproaches against his wife on account of her marital frigidity and childlessness” (Freud, 1901/1974^2^). A patient of the first author, troubled by the increasingly feminine ways of his 4-year-old daughter, spoke of his “fear year old daughter.” In a footnote of *Psychopathology of everyday life*, which only appears in the French translation, Freud (1901/1953, p. 239) holds: “We think we are generally free to choose words and images to express our ideas. But a closer observation shows that it is often considerations extraneous to the ideas that decide this choice and that the form in which we mold our ideas often reveals a deeper meaning, which we do not realize ourselves. (…) some of these images and ways of speaking are often allusions to subjects which, while remaining in the background, exert a powerful influence on the speaker. I know someone who, at one time, continuously used, (…) the following expression: “When something suddenly crosses the head of someone.” Now I knew that the man who spoke in this way had recently received the news that a Russian projectile had passed through the field-cap which his son, a fighting soldier, had on his head^3^” The same would hold true in dreams, in symptoms and in psychotic delusions. For symptoms, e.g., Breuer and Freud (1893-95/1955, p. 216; Italics added) indicate that there is an irrational “symbolic relation between the precipitating cause and the pathological phenomenon” which, indeed, is “often based on the *most absurd similarities of sound and verbal associations*.”

All this has fuelled the interest for phonology in the unconscious processing of language in some of the studies of (late) Howard Shevrin and his team. For example, in a study with subliminal priming of reversible words (such as “sleep”/”peels”) a tachistoscopic paradigm was used to test for the unconscious recognition of the reversed readings (Klein Villa et al., 2006). In this experiment, a 1 ms reversible target, such as e.g., the word “dog,” was followed by a semantically related target and a distracter. In the forward condition, the related target was a straightforward semantic associate of “dog” (e.g., “canine”) while in the reverse condition, it was a semantic associate of the reversed word (i.e., “god,” e.g., “angel”). There was only straightforward priming with supraliminal presentation. But in subliminal presentation of the targets, there was semantic priming in *both* forward and reverse conditions in anxious participants. At the other hand, and strikingly, with low self-evaluation of anxiety there was a proportionate avoidance of the semantic associate in both conditions subliminally.

These observations are in line with previous results with subliminal priming at 1 ms priming (unmasked) and with a stimulus detectability parameter of d' not different from zero. Snodgrass and colleagues (Snodgrass et al., 2004; Snodgrass and Shevrin, 2006) have extensively argued that under these stringent conditions, priming effects only revealed themselves as an interaction effect with personality factors. Indeed, Shevrin (1992) has suggested before that, whereas at a conscious level, personality factors generally do not interfere with cognitive tasks, this becomes a different matter at a subliminal, or unconscious, level. In the Klein Villa study, it was found that participants with low trait anxiety did not show an absence of semantic priming, but an inhibition, meaning that they chose the distracter at higher levels than chance. Likewise, Snodgrass et al. (1993) had their participants to identify one out of four words, known to them, presented at 1 ms. They were are asked to use one of two strategies: in the *look* strategy, subjects were instructed to attend carefully to the visual field and look hard for any trace of the stimuli; in the *pop* strategy, subjects were urged to allow one of the four stimulus words to pop into their minds—to say whichever of the four words comes to mind. Subjects were also asked which of the two conditions they preferred; they were called “lookers” or “poppers” according to their preference. The 1993 experiment was replicated both in the Shevrin lab (Snodgrass and Shevrin, 2006) and by Van Selst and Merikle (1993). The main consistent finding in the original experiment and in its replications, then, is that “poppers” facilitated slightly in the pop condition, giving more correct answers than chance level (25%), while “lookers” did better than chance in the look condition and performed significantly below chance in the pop condition (for meta-analysis, see Snodgrass and Shevrin, 2006). This pattern of results illustrates an important qualitative characteristic of unconscious processes, namely that inhibition can occur in situations in which conscious perception would characteristically produce facilitation.

In the present study we wished to further investigate the subliminal processing of word phonology at the same stringent priming conditions with d' = 0. In the experimental trials, one of the targets was a direct phonological reversed form of the prime (e.g., “door” and “road”), while the other target was a non-related distracter (e.g., “lung”). Moreover, to be able to disentangle phonology and orthography, the phonological targets were perfect phonological reversed forms, though they were orthographically dissimilar (e.g., “lakes” and “scale,” “talk” and “caught,” “moan” and “gnome”). In contrast to the Klein Villa et al. (2006) study, both the prime and the two targets were presented at 1 ms. Though the participants did not see anything during the whole sequence, they were urged to make a choice as to which target they thought was most similar to the prime (after having had fully visible practice sequences): they said “one” for the upper choice and “two” for the lower choice. Moreover, we measured Event Related Potentials (ERPs) to explore for physiological markers of the recognition of subliminal phonological similarity. Indeed, Shevrin and colleagues had shown that subliminal stimuli elicit ERP patterns that are structured similarly to supraliminal ERP patterns at all electrodes, be it at a lesser amplitude (Shevrin and Fritzler, 1968; Shevrin et al., 1992, 2010, 2013; Bernat et al., 2001a,b; Silverstein et al., 2015). Since we wanted to verify if participants were unconsciously able to detect the phonological similarity between pairs of reversed words, we focused in particular on a negative N320 component sensitive to phonology and known to peak between 300 and 450 ms after target presentation in phonological oddball paradigms in particular.

Indeed, previous work that has investigated participant response to consciously presented, phonologically similar stimuli and their associated brain indices has found that the N320 is indicative of phonological processing. For example, Bentin et al. (1999), using a visual word presentation paradigm, proposed a rhyme task in which the targets were words or pseudowords rhyming with the word *vitrail*, with orthographically possible endings being “aille,” “ail,” “aye,” or “aï.” Nontarget stimuli did not rhyme and elicited a negative potential peaking at about 320 ms after stimulus onset, which was called N320. Bentin et al. (1999) concluded that the N320 could represent an early lexical or prelexical process of grapheme-to-phoneme-to-phone translation. In addition, Grossi et al. (2001) also used a visual rhyme task with orthographically dissimilar rhyming pairs (ex. “juice” and “moose”) and found a negative deflection which began between 250 and 300 ms, peaked between 300 and 400 ms, and which was larger for non-rhyming than rhyming targets. They concluded that this slow wave asymmetry “may reflect the allocation of resources to areas specific to phonological decoding of written words” (Grossi et al., 2001, p. 621). Simon et al. (2004) and Simon et al. (2006) instructed participants to passively attend to the visual presentations of words and pseudowords. They found that adult skilled readers in French displayed a specific component (N320) with a left occipito-temporal scalp distribution. This component was implicated in phonologic transcription and taken to mark the use of grapheme-phoneme conversion. Interestingly, Proverbio et al. (2004) have proposed a relation between this negativity in response to written word presentation and an error mismatch negativity in response to auditory word presentation occurring at 270–300 ms, i.e., the “phonological mismatch/mapping negativity” (PMN; Connolly et al., 1995, 2001; D'Arcy et al., 2000; Dehaene-Lambertz et al., 2000) which is selectively sensitive to phonology. It is thought to index a response that reflects phonological processing in all relevant circumstances but is larger when the analysis of an incoming acoustic signal mismatches phonemic expectations (Newman et al., 2012, p. 145). In conclusion, the N320 component, both in the visual rhyme and word judgement tasks (Bentin et al., 1999; Grossi et al., 2001; Simon et al., 2004, 2006) and in the acoustic tasks, is thought to reflect mismatch with phonological expectation, which in visual tasks specifically requires a higher activation of the grapheme-to-phoneme conversion process.

In the present research, there was only phonological similarity in one of the targets of the experimental trials while in control trials, none of the targets was similar to the prime. Consistent with previous findings, we expected that this design would produce a *subliminal* N320-effect if phonological mismatch was recognized in the control trials as compared to the experimental trials and that this effect would depend upon personality factors. Moreover, if a correlation between the ERPs and the behavioral choices made by the participants is found, this would suggest that, though the participants are convinced they made their choices in a totally arbitrary way—since they couldn't see anything consciously—their choices were nevertheless informed by processes they are unaware of.

## Materials and Methods

### Participants

Thirty-two right-handed paid participants took part in the study. They had a mean age of 21.8 (range 18–33, *SD* = 3.07), 22 were women, all had vision correctable to 20/20 and all reported no history of neurological or psychiatric problems. Participants were recruited through a newspaper advertisement. One participant claimed he sometimes could see the stimulus. Accordingly, he has the highest score of phonological choices of all participants (20 out of 30) and a fairly high detectability score (d' = 0.32). These atypical scores make him an outlier; therefore he was excluded from data-analysis. Therefore, the *N* of this experiment is 31.

### Materials: Apparatus, Masking Technique and Word Stimuli

The stimuli were presented on 4 X 6 inch white cards in the two fields of a three-field Gerbrands Model T3-8 tachistoscope. The first field was the prime, the second field was the target field and the third field was used as a fixation field. Luminance levels for the stimulus fields, as well as the ambient light level in the subject chamber, were set at 5 foot/lamberts luminance and the duration for the subliminal presentation was 1 ms.

A prime word was followed by a target card with two target alternatives, a phonological choice and a non-related choice. The two target alternatives were at equal distances above and below the position of the fixation point and counterbalanced over the different items. All words were one-syllable in length. The phonological target alternative was the exact phonological reversed form of the prime word (Webster dictionary), although it was orthographically dissimilar. Sometimes the orthographic dissimilarity was substantial such as e.g., “nice-sign,” “chance-snatch,” “moan-gnome,” “caught-talk,” sometimes the orthographic dissimilarity was very limited such as e.g., “boss-sob,” “spill-lips,” “till-lit,” “sap-pass” (see Table S1 for the full list with the phonological transcriptions).

The orthographic similarity between each target word and the prime word was calculated using Weber's graphemic similarity index (Weber, 1970): OS = 10 ^*^ [((50^*^F + 30^*^V + 10^*^C)/A) + 5^*^T + 27^*^B + 18^*^E] with *F* = Number of common bigrams in both words, *V* = Number of common inverted bigrams, *C* = Number of common letters, *A* = Mean number of letters in both words, *T* = Proportion of number of letters in the shorter words with respect to the longer word, *B* = Common starting letter, and *E* = Common ending letter.

Each subject is presented 30 experimental trials and 30 control trials in a random order. For the construction of the control trials we divided a total of 60 triads in two lists. A subject is presented either the 30 triads of list 1 (experimental trials) and 30 recomposed triads of list 2 (control trials) or vice versa (list 1 serves as the control list, list 2 is the experimental list); this alternation is rotated over the subjects. The list of control trials is composed by pseudo-randomly attributing the target words to the primes to which they don't belong in within the same list.

The behavioral dependent variable is the number of phonological choices. In the experimental condition, the phonological choice is the target choice, which is the phonological reversed version of the prime; in the control condition the “phonological choice” is the target choice, which is the phonological reversed version of the original prime it belonged to. The experimental effect is the difference between the number of phonological choices in the experimental condition (out of 30) and the number of phonological choices in the control condition (out of 30).

### Experimental Procedure

#### Main Experiment

After a brief introduction to the laboratory, participants completed an informed consent statement; informed consent and all procedures were approved by the Institutional Review Board of the Department of Psychiatry of the University of Michigan. Electrodes were attached prior to seating participants in a sound-proof, electrically shielded, temperature-controlled booth.

In order to familiarize the participants with the unusual experimental task, we presented a series of practice stimuli prior to the experiment proper. There were no obvious similarities between prime and targets in any of the practice trials to avoid inducing a systematic search for any type of similarity, semantic, phonological or orthographic; participants were not informed of the type of similarity used for the experimental presentations. First, a fully supraliminal practice trial was presented “YEAR” (prime; 500 ms) followed by “HOLE NIECE” (targets; 4,000 ms) in order to familiarize the participant with the experimental task: “*give the choice that in your opinion is most similar to the first word*.” Second, a second practice trial “JUICE” followed by “BELT EARN” was presented 4 times subsequently whereby the presentation times of both prime and targets were gradually decreased from 30 to 10, then to 5 and finally to 1 ms in order to familiarize the participant with the subliminal presentations. Participants had to say “one” if they picked the upper word and “two” for the lower word (no numbers were indicated on the card). Third, a third practice trial “AGE” followed by “DOLL NERVE” was presented directly at 1 ms for both prime and targets, in order to familiarize the participant with the subliminal experimental condition. Finally, two last practice trials “BARN” followed by “MOLD QUICK” and “CALM” followed by “PINK STOVE” were presented at 1 ms for both prime and targets and including the EEG measurements (as indicates below) to familiarize the participant with the full experimental conditions.

The main experiment consisted of 60 subliminal random presentations. For all trials, participants were instructed to remain as still as possible, focus on the fixation point, pay attention, and keep eye blinks to a minimum during each stimulus presentation. This instruction was repeated periodically during the experiment.

The stimulus delivery sequence was as follows:


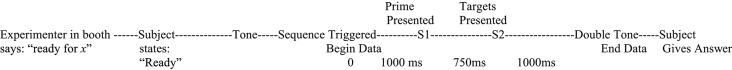


First, the experimenter in the booth, who was blind to the stimulus content and experimental hypotheses, called “ready for x” with x being the number of the trial. This was the signal for the participant to focus his/her eyes on the fixation point. When the subject was ready for the sequence to begin, he/she says “ready.” The experimenter outside the booth, who was also blind to the stimulus content, monitored the EEG recordings for possible eye blinking or excessive muscle movement. If the record appeared free of artifacts, the EEG experimenter pushed a button producing a first tone, which preceded the triggering of the sequence. Participants were told to anticipate a sequence of quick presentations of words on the screen that they may or may not be able to see, and that their responses, including their brain responses, would be monitored. The sequence consisted of 1,000 ms fixation point, after which the prime word was presented for 1 ms, followed by the fixation field again for 749 ms; then the target card for 1 ms, again followed by the fixation field for 1,000 ms. After that came a double tone, signaling the end of the sequence and of the data collection period. This was the signal for the subject to relax, move, blink and give his/her answer. Records contaminated with artifacts (determined by online visual inspection; ≤ 5% per participant) were rejected and the trial was presented again in the next cycle. Participants were not informed when trials are retaken vs. advancing normally to the next trial. The final source of variability was the time between the double tone and the next single ready tone. Most frequently this was about 5 s, which is the time it took for the experimenter in the booth to change the stimulus cards.

#### Detection Control Task (Conscious Perception Index)

A 64-item, forced-choice detection task was administered at 1 ms and 5 foot/lamberts luminance (the same conditions as during the experiment) to determine if stimulus presentations met the criteria for the objective detection threshold (Snodgrass et al., 2004; Snodgrass and Shevrin, 2006). The sequence of the cards was the same: a first prime card was presented first and then a second target card with two words, one above and one under the fixation point. In half of the trials the cards with the words (both prime and targets) had been replaced with plain blank cards. The 64 trials were the same for all participants and consisted of 32 blank primes and blank targets and 32 word primes and word targets. The 32 word *primes* consisted of 16 word primes out of each list; the 32 word *targets* consisted of 8 targets to the primes and 8 random targets for each list (see Table S1). The subjects' task was to state after each presentation what they believed had been presented, the word or blank cards, and to keep their responses roughly equally divided between the two choices.

#### Supraliminal Control Experiment

To control for the fact that the participants were able to see the phonological similarity when the stimuli were presented fully consciously, they completed the same tasks as in the main experiment with the same stimuli in the same order, but supraliminally on pen and paper, after the detection task. Participants were simply given the same triads on a paper sheet and circled the choice they thought was most similar to the prime word. (Due to time constraints for the participants' comfort, we could not add a supraliminal part to the tachistoscope-EEG part of the research).

#### Social Desirability Scale (SDS)

Next, they completed the Personal Reaction Inventory (Crowne and Marlowe, 1960). This 33-item true/false scale is a social desirability scale (SDS) which measures the need for social approval, and the readiness or reluctance to report negative emotional states. It evaluates to what extent people can admit so-called unacceptable but nevertheless quite universal truths about human functioning—e.g., “I have never deliberately said something that hurt someone's feelings.”

### Physiological Measurement Apparatus

The recording sites were F_P1_, F_P2_, F_3_, F_Z_, F_4_, C_Z_, T_3_, T_4_, T_5_, T_6_, P_Z_, P_3_, P_4_, and EOG according to the International 10–20 Electrode Placement System. Next to the traditional midline electrodes Fz, Cz, Pz, all temporal electrodes (T3, T4, T5, T6) and parietal electrodes P3 and P4 were added, as the research was linguistic in nature; frontal electrodes were of interest since phonology also implies frontal circuits (such as Broca's area). Electrode caps (BIOPAC Systems, Inc.; CAP100C; medium 54–58 cm and large 58–62 cm) were used: this Lycra stretch cap holds 19 imbedded tin electrodes closely to the subject's head; electrodes are pre-positioned in the international 10/20 montage. When the electrode cap is in place, electrode sites were cleaned with a mild abrasive solution and EEG recording gel was injected into each electrode with a blunt-tipped syringe. All electrodes were referenced to linked ears with a mastoid ground. Electrode impedance was <3 kΩ. Eye activity was monitored by electrodes placed on the outer canthus and suborbital ridge of the right eye.

All signals were collected utilizing a Grass Model 8-24D polygraph linked to a Macintosh computer. Signals were digitized at 250 Hz through a National Instruments NB-MIO-16X A-D board controlled by LabVIEW 2.2 (National Instruments) software, then stored in computer files for off-line analysis. Signals were analog filtered online through Grass Model 8A5 AC amplifiers with a low-pass frequency of 100 Hz, and a high-pass frequency of 0.1 Hz. ERPs were sampled for 2,750 ms, including a 1,000-ms prestimulus interval. For the N320 component, the signal was filtered at 9 Hz.

Because the tachistoscopically presented subliminal 1 ms stimuli (black print on a white background) are preceded and followed only by a fixation field (black dot on a white background) of equal luminance, there was very little disturbance in the visual field. This resulted in an ERP waveform which was substantially smaller in amplitude and “noisier” than conventional ERP waveforms to supraliminal stimuli.

### Data Reduction

Individual ERP trials were averaged across the experimental and the control trials. Component windows were defined based on grand average ERP wave forms across all presentations and participants (Hoormann et al., 1998). This process was completed within participant and electrode. Component values from the averages in the experimental and control items were then compared statistically. Baselines were calculated as the mean amplitude from 500 to 988 ms prior to prime presentation. The time interval for N320 was 300–372 ms locked to the targets (see introduction). Then an automated computer program was used to find the most negative peak within this window (300–372 ms) for each experimental and control averaged waveform. A similar pattern of results was fond using filters of 25 Hz.

### Statistical Analyses

The experimental ERP component-effect is the difference in amplitude between the component in the experimental condition (where one of the targets is the phonological reversed form of the prime) and the component in the control condition (where none of the targets are related to the prime). For the negative N320 component, the literature review (see above) leads us to expect that the experimental component would be smaller than the control component, resulting in a positive difference or experimental effect. A negative difference or effect means that the experimental component is larger in (negative) amplitude than the control component. Due to the relatively low *N* and the exploratory nature of the ERP analyses, which included other components that are not relevant to the current research question and thus not reported here, we have not adjusted alphas for multiple comparisons leaving significance values at *p* < 0.05.

## Results

### Behavioral Results

#### Subliminal and Supraliminal Forced-Choice Tasks

In the subliminal priming condition, the mean number of phonological choices was 13.9 ± 0.4 (± Standard Error of the Mean) out of 30 in the experimental condition, and 15.0 ± 0.5 out of 30 in the control condition. There was no principal behavioral effect, experimental vs. control, on the number of phonological choices picked by the participants, though there was a tendency to pick the non-related choice (*p* = 0.068; see Table 1). In the pen-and-paper supraliminal condition, there was a clear choice for the phonologically similar words in the experimental condition with means of 22.7 ± 0.9 in the experimental vs. 14.8 ± 0.5 in the control condition (*p* < 0.001). Therefore, clearly participants are able to recognize phonological similarities even if in the present research we used phonological reversed forms. A two-way repeated measures ANOVA [condition (experimental, control) by threshold (subliminal, supraliminal)] shows a significant interaction effect of condition x threshold [*F*_(1, 30)_ = 65.7; *p* < 0.001; η_*p*_ = 0.69].

**Table 1:** Main behavioral effects: mean number of phonological choices ± SEM (out of 30; *N* = 31).

	**Experimental**	**Control**	***p***
Subliminal	13.9 ±.4	15.0 ±.5	0.068
Supraliminal	22.7 ±.9	14.8 ±.5	< 0.001

#### Control for Subliminality

Detection d' was 0.044 ± 0.047 with a range from −0.40 to 0.74 (95% confidence-interval: −0.0523 to 0.1408) and was not significantly different from zero (*p* = 0.357). Moreover, the participants were asked at several times during the experiment if they saw something: (1) after each practice trial; (2) halfway the experiment and (3) after the detection experiment. Finally, on debriefing they had to indicate if at any point during the experiment they noticed something. For the 1 ms presentations none of the participants indicated seeing something at any point (except for the excluded participant). Altogether, these data confirm that the energy mask effectively precluded conscious recognition of stimuli at 1 ms exposure.

Note that there was no correlation between the behavioral effect (net number of phonological choices) and the d' (*r* = 0.05; *p* = 0.783; see Figure S1).

#### Social Desirability Scores

**Figure 1:**
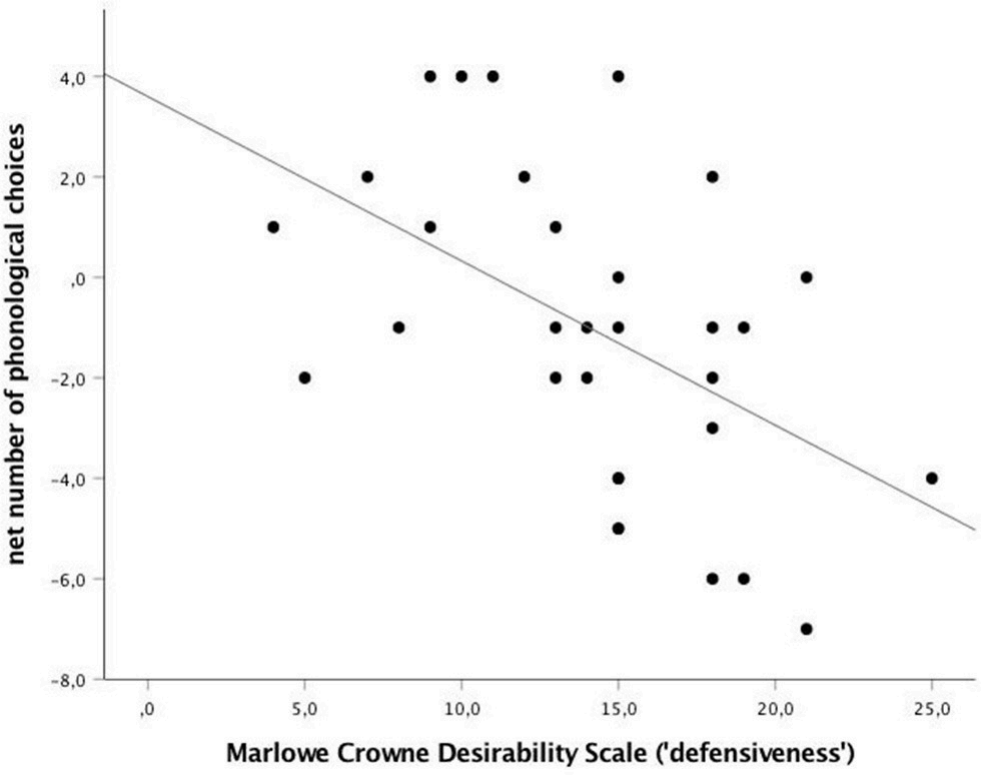
Low defensive participants make phonological choices, high defensive people avoid them. Net number of phonological choices (experimental–control) in function of the Crowne-Marlowe Social Desirability scores (*r* = −0.51; *p* = 0.004; *N* = 31). The less “defensive,” the more net phonological choices made by the participant in a fully subliminal priming experiment with tachistoscopic presentation of both prime and target at 1 ms; the participant's task was to pick the target choice which was most similar to the target, choosing between a phonological reversed target (e.g., “DOOR” and “ROAD”) and a non-related target (e.g., “LUNG”).

The mean SDS-score was 14.3 ± 0.9 (out of 25) with a range from 4 to 25. Subliminally, there is a substantial correlation between the experimental effect (net number of phonological choices) and the social desirability score: the higher the SDS-score, the smaller the experimental effect (*r* = −0.51; *p* = 0.004). Only at low SDS is there a net positive experimental effect with more phonological choices in the experimental than in the control conditions. At mean or high SDS there is a negative experimental effect, i.e., phonological choices in the experimental conditions are significantly less frequently chosen than chance, i.e., they are avoided (see Figure 1). Supraliminally, no correlations were found between the net number of phonological choices and SDS (*r* = 0.16; *p* = 0.397).

### No Difference Between Experimental and Control N320 Parameters

A schematic topographical plot based upon the amplitude values given in Table S2 shows that N320 is largest at midline electrodes Fz, Cz, and Pz and is also more left than right-lateralized. Based on this, but also relying up on theoretical reasons (Bentin et al., 1999; Proverbio et al., 2004; Simon et al., 2004, 2006; see Discussion) given we measure here ERPs to subliminal stimuli, we regrouped two components, a midleft component (average of electrodes F_P1_, F_3_, F_Z_, C_Z_, T_3_, T_5_, P_Z_, and P_3_; −0.53 ± 0.13 μV) and a right component (average of F_P2_, F_4_, T_4_, T_6_, and P_4_; −0.35 ± 0.14 μV; *p* = 0.001).

A repeated measures ANOVA for the N320 amplitudes (in μV) with localization (midleft vs. right) and condition (experimental vs. control) as factors gave a highly significant localization contrast [*F*_localization__(1, 30)_ = 14.531; *p* = 0.001]. However, there was no main effect between experimental and control trials [*F*_condition__(1, 30)_ = 0.090; *p* = 0.776] and also no interaction effect. A repeated measures ANOVA for the N320 latencies (in ms) with localization and condition as factors (same as above) gave no localization contrast [*F*_localization__(1, 30)_ = 0.007; *p* = 0.934], no main effect between experimental and control trials [*F*_*condition*__(1, 30)_ = 2.714; *p* = 0.110] and also no interaction effect. As concerns the condition main effect, there is a non-significant tendency for the N320 to come earlier in the control vs. the experimental trials (335 ms ± 4 vs. 343 ms ± 4; 2-tailed). At the midleft electrodes the N320 thus peaked between 304 and 376 ms (with a μ = 339 ± 4 ms) and with amplitudes between −0.31 and −0.74 μV (with a μ = −0.47 ± 0.12 μV).

### The N320 Characteristics Significantly Predict the Behavioral Response Choice

**Figure 2:**
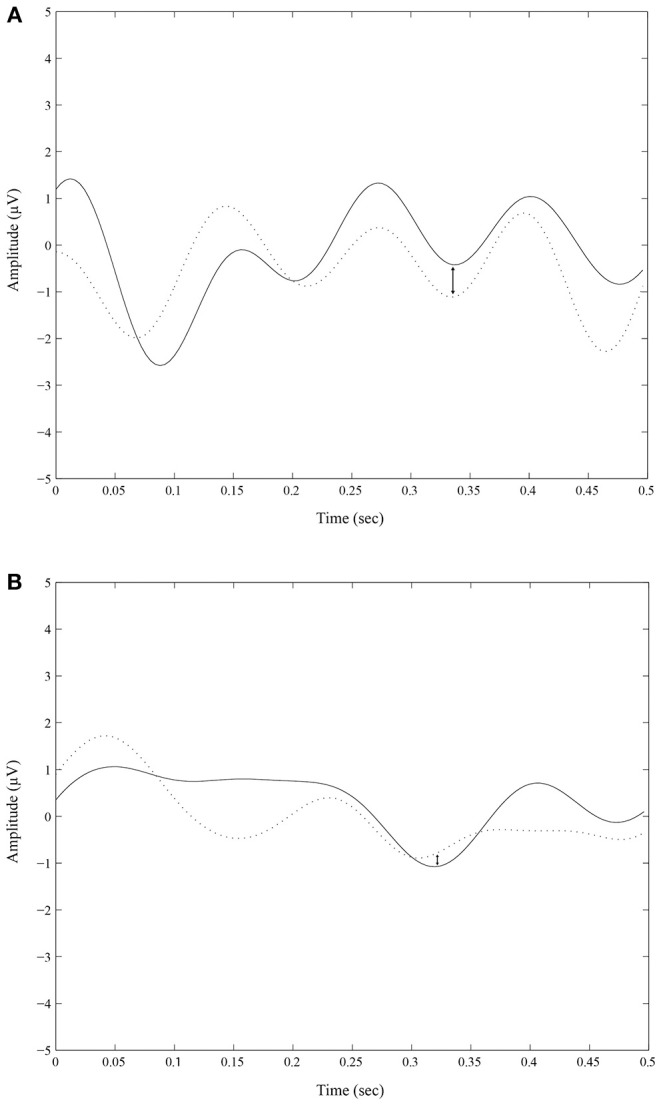
ERP traces for a participant with a high number vs. a low number of phonological choices. Experimental (solid line) vs. control (dotted line) N320 amplitudes at the mid-left of the brain (average of F_P1_, F_3_, F_Z_, C_Z_, T_3_, T_5_, P_Z_, and P_3_ electrodes) **(A)** for a participant with a high net number (4) of phonological choices and **(B)** for a participant with a low net number (−7) of phonological choices (right). The N320 waves are indicated.

Even if there is no main effect of condition (experimental vs. control), there is an interaction effect between the N320 amplitude difference (experimental minus control) and the behavioral choices the participant picks ca. 1 s later: the larger the N320 amplitude effect at the mid-left of the brain, the more likely the participant will pick the phonological target response (*r* = 0.43; *p* = 0.01). Two ERP traces are shown in Figure 2; ERPs may appear unusual due to the extremely brief exposure durations and no backward masking, but Bernat et al. (2001a) have shown that they possess the same component structure as supraliminal ERPs. Note that there is no correlation at the right of the brain (*r* = 0.24; *p* = 0.197).

**Figure 3:**
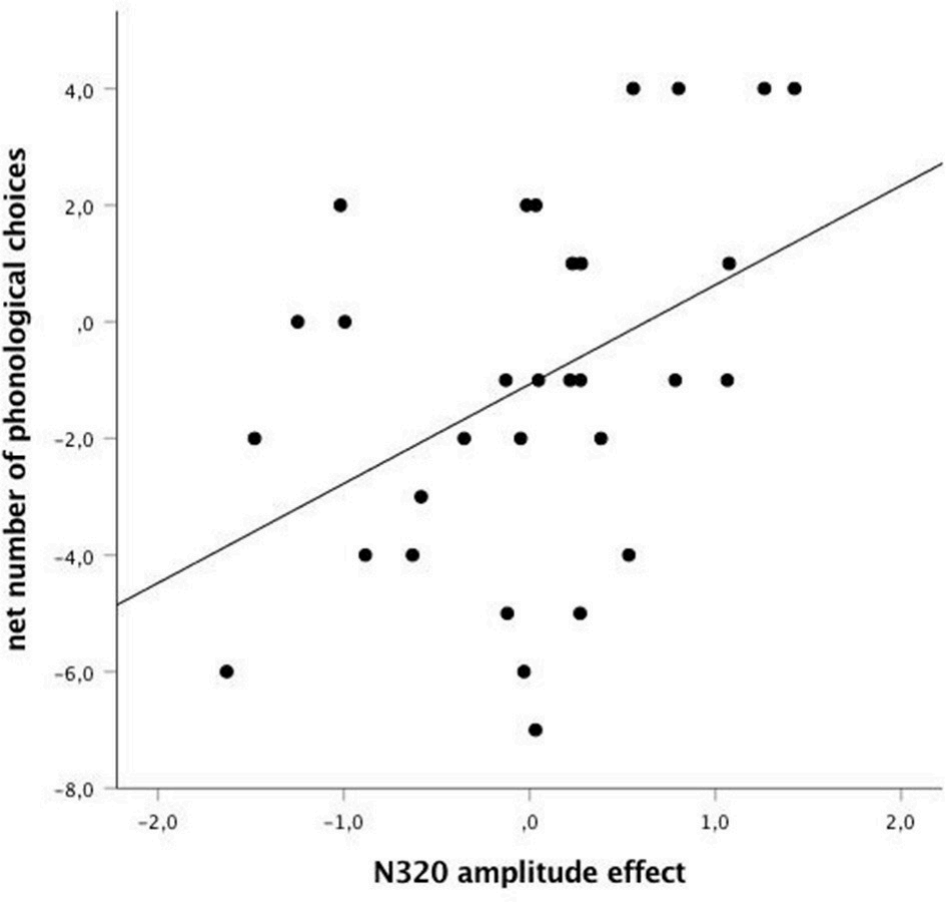
Net number of phonological choices by N320 amplitude effect (experimental—control) at the mid-left of the brain (average of F_P1_, F_3_, F_Z_, C_Z_, T_3_, T_5_, P_Z_, and P_3_); *r* = 0.43; *p* = 0.017); *N* = 31. The more positive the N320 amplitude effect (i.e., the more negative the N320 amplitude in control trials as compared to experimental trials), the more net phonological choices.

**Figure 4:**
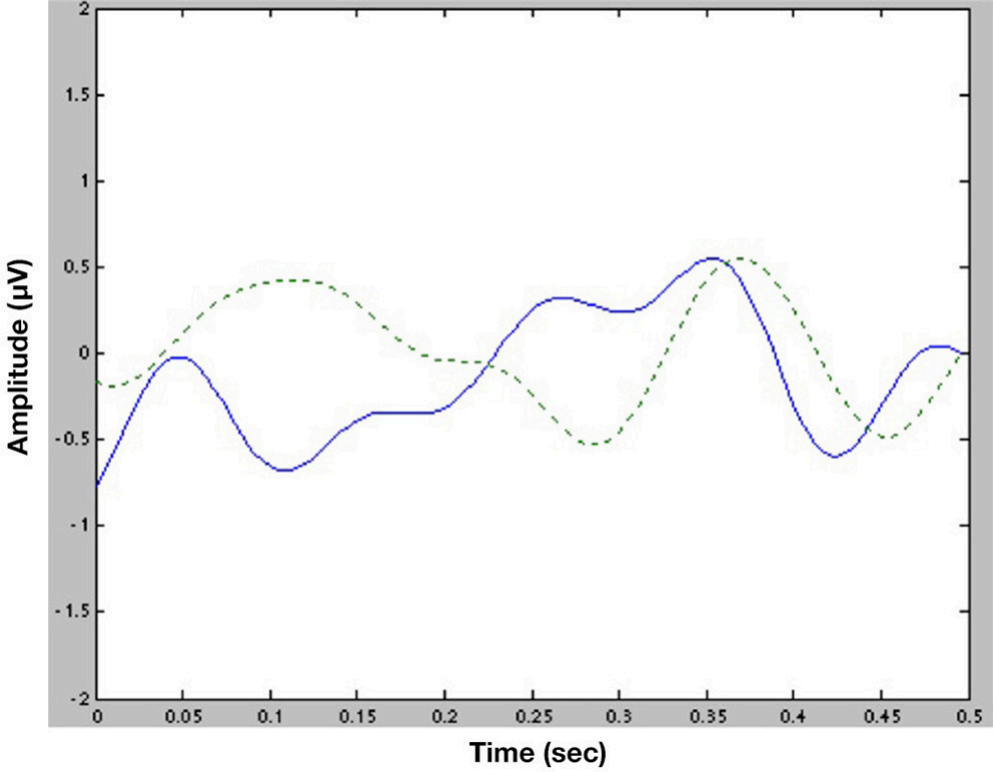
Average ERP traces (experimental minus control) at Cz for subjects with a positive behavioral effect (μ = 2.5; *N* = 10; solid line) vs. for subjects with a negative behavioral effect (μ = −3.1; *N* = 19; dotted line). The higher the difference between experimental and control at N320, the more phonological choices ca. 1 s after the N320.

In other words, the more the N320 amplitude effect is positive (experimental more positive than control), the more the participant chooses the phonological response; the more the N320 amplitude effect becomes negative (experimental less positive than control), the more the participant will pick the non-related target response (see Figures 3 and 4). In other words still, the more the participant show electrophysiological signs of recognizing the phonological reversed word subliminally, the higher the probability that he/she will pick that choice (even if to the participant, he/she is just saying “one” or “two”).

Note that there is also a significant interaction of the N320 amplitude effects with social desirability. Indeed, the midleft N320-amplitude effect correlates negatively with MC social desirability (*r* = −0.49; *p* = 0.005; see Figure S2). The correlation is also significant at the right of the brain (*r* = −0.39; *p* = 0.033). The correlation results are logical since as we have shown that the N320 amplitude effect correlates with the behavioral effect (see Figure 3) and that the behavioral effect correlates with the SDS.

### Item Analysis: The Orthographic Similarity Between Prime and Phonological Target Does Not Influence Either the Behavioral Choices or the N320 Amplitude

The phonological targets are composed of exactly the same phonemes as the primes but in a reversed order; however they are never totally orthographically similar to the primes and sometimes they are, in fact, quite dissimilar (ex. talk/caught). However, as is shown in the Table S1, they are still more orthographically similar to the primes than are the distracter items. Therefore, we do not know if the effect measured is mainly carried by the phonological similarity or by the orthographic similarity between the prime and the phonological target. To disentangle both effects we have calculated an orthographic similarity (OS) index using Weber's graphemic similarity index (see Table S1) between prime and phonological target. The orthographic similarity between the prime and any of the targets does not affect the chance of the phonological target to be chosen: there are no correlations between the net number of phonological choices and the orthographic similarity neither subliminally nor (*r* = 0.062; *p* = 0.639) supraliminally (*r* = 0.020; *p* = 0.881). Moreover, Table S3 shows that correlations between the N320 amplitudes in the experimental condition with the OS between the prime and the phonological target are mostly negative, but we find marginally significant effects at the two prefrontal sites and at *F*_3_ exclusively. In other words, at these electrodes exclusively, orthographic similarity between prime and phonological target might slightly and non-significantly contribute to the N320 reduction upon presentation of the phonological target.

## Discussion

### There Are No Main Effects

No main effects were found: there was no significant behavioral choice for the phonological alternative in the subliminal condition and we did not find a significant difference for the whole group between experimental N320 and control N320. The 1 ms-tachistoscope presentations were very stringent conditions of subliminality, said to be at the “objective detection threshold” (Snodgrass et al., 2004; Snodgrass and Shevrin, 2006). It might be feared that this stimulus intensity is too low for anything to happen. However, Shevrin and colleagues have repeatedly shown that these kind of subliminal stimuli elicit ERP patterns that are structured similarly to ERP patterns evoked by supraliminal stimuli at all electrodes, be it at a lesser amplitude (e.g., Shevrin and Fritzler, 1968; Shevrin, 1973; Bernat et al., 2001a,b; Silverstein et al., 2015). Moreover, in previous research, it has rarely been possible to observe main effects. For example in the “pop-look” study (see above; Snodgrass et al., 1993), though overall identification was at chance, it was found consistently that poppers and lookers did better than chance in their preferred condition and that lookers performed significantly below chance in the pop condition. In the study by Klein Villa et al. (2006), again, there was no main effect but there was a significant interaction effect with trait anxiety, with facilitation for semantic priming in high trait anxiety and avoidance in low trait anxiety. Because of these bidirectional tendencies operating in function of personality, there is an absence of main effect (for a review on unconscious inhibition, see Bazan and Snodgrass, 2012). To explain the avoidance results, we suggested the implication of defensive operations. For the pop-look study Snodgrass and Shevrin (2006, p. 63) proposed: “when utilizing the strategy congruent with their preference, perhaps participants unconsciously allow this activation to influence their response, elevating performance above chance. In contrast, when utilizing the incongruent strategy, such influences are unconsciously rejected and below-chance performance ensues.” The looker inhibition then “might reflect a simple form of unconscious defense […]. Along these lines, lookers consistently expressed a strong preference for activity and control, explaining that they disliked ‘doing nothing' as the pop instructions required. Obliging lookers to relinquish conscious control with pop instructions might instantiate a mildly conflictual situation, producing inhibition, whereas more congenial look instructions would not, yielding facilitation.” For the Klein Villa et al. (2006) study we proposed that a defensive organization may imply an effective inhibition of word associativity (Freud, 1895/1966, 1900/1958; see also Bazan, 2006). Anxiety interferes with the organization of defense so that people with structurally higher levels of anxiety might not be able to defend as efficiently and show associative facilitation, explaining the results. Alternatively, it might also be that people, who on a paper-and-pen questionnaire claim to be usually less anxious, are more defensive than people who admit more anxiety experience and thereby inhibit associativity better. This is in line with Rapaport (1967) who posited that personality and its use of defensive organization can have a powerful regulating control over cognition.

### N320 Amplitude Predicts Behavioral Choices

Our main finding, then, is that the behavior of a negative wave, peaking around 340 ms at midleft locations, correlates with the behavioral choice of the participant more or less 1 s later. In other words, the more the amplitude effect is positive (experimental more positive than control), the more the subject chooses the phonological target; the more the amplitude effect is negative (experimental more negative than control), the more the subject chooses the non-related target. Because of this behavior we propose that this negativity could correspond to the N320. Indeed, this N320 has been described as being mobilized specifically when phonological decoding is at stake: the N320 component would represent the allocation of resources to areas specific to phonological decoding of written words (Grossi et al., 2001) or recoding of written words to phonemic representations (Bentin et al., 1999; Simon et al., 2006). This, then, is especially needed when the targets are phonologically different from the prime, i.e., more for the control than for the experimental targets. The more positive the difference between experimental and control N320 amplitude i.e., the more the participant recognizes the phonological similarity, the more he chooses the phonological target. Note that orthographic similarity does not influence the behavioral choices and that there is no significant contribution of orthographic similarity to the behavior of the experimental N320.

As concerns the spatial localization of this negativity over the scalp, it is best observed at the midline and left of the brain and the correlations with subsequent behavioral choices are especially high at midleft electrode sites. These results make sense with the phonologic linguistic decoding needed for the task. These results are also in some aspects comparable to e.g., those of Bentin et al. (1999) where the N320 was most evident over the temporal and temporo-parietal regions bilaterally but significantly larger over the left than over the right hemisphere and with those of Simon et al. (2004, 2006) who found a left occipito-temporal scalp distribution. In the present study there is no shift toward the back of the brain for the N320 (see Table S2). Proverbio et al. (2004) had shown that there is an antero-posterior topographic dissociation for N320 whereby access to the phonemic representation of letter strings would activate the left occipito-temporal regions for reading words and pseudowords and frontal regions for reading letter strings. In the present research, participants read both distinct words (e.g., the prime word and the non-related target) and words which share phoneme strings even if in reversed orders (the prime and the phonological target). This would fit well with our observations that N320 appears in a comparable way at frontal and parietal recording sides.

It thus appears that a phonological mismatch negativity, the N320, characterized for (visually presented) words in supraliminal conditions, also plays a comparable role in subliminal conditions. The subliminal conditions in the present experiment are very rigorous: the d' is at zero for the whole prime-target sequence and there is no correlation between the behavioral choices and the d', excluding any contribution of conscious detection to the results (see Snodgrass et al., 2004; Snodgrass and Shevrin, 2006).

Moreover, it appears that unbeknownst to the participants themselves, who are convinced that they are making arbitrary choices, saying “one” or “two” randomly, nevertheless a pattern can be seen in their behavioral responses, as those can be significantly predicted by their N320 waves. The amplitude of the N320 happening at the mid-left of the brain ± 340 ms after subliminal target presentation can significantly contribute to predicting the behavioral target choice the participant will make, ± 1,000 ms after target presentation, i.e., at the earliest ± 650 ms later than the N320 wave, while all the same the participant is having the experience of a totally arbitrary choice. Hence, we believe evidence is given for the association of physiologically measurable unconscious processes with subsequent conscious behavioral choices.

### High SDS-Scorers Avoid Ambiguous Choices Unconsciously

While in the supraliminal experiment, participants clearly recognized and choose the phonological target, a majority (19 out of 32) of the participants choose a majority of non-related targets in subliminal conditions. In fact, the number of phonological choices is almost significantly less-than-chance level in the experimental condition (*p* = .068). Moreover, when we regress these results with the level of social desirability, measured with the Personal Reaction Inventory (Crowne and Marlowe, 1960) we find that there is an inverse relationship between this social desirability and the participants' responses: participants with low SDS give more phonological responses while participants with high SDS give more distracter responses. As these distracter items have no systematic relationship with the prime, neither phonological nor semantic, it must be assumed that a positive choice for the distracter is, in fact, a negative choice for the phonological choice, i.e., people with high SDS *avoid* the phonological target. How comes participants choose the non-related item when there is a phonologically similar choice?

In fact, their behavior is in average “logical” with their electrophysiology: the experimental N320 is more negative than the control N320, which, on the basis of the literature in supraliminal research, is indicative for the detection of more phonological dissimilarity in the experimental targets than in the control targets, hence the non-related target is picked. It is strange, though, that the phonologically similar target could behave subliminally as less similar than the control target. However, if we remember that the N320 component represents the allocation of resources to phonological decoding of written words, we must infer that these participants, who “avoid” the phonological target, mobilize a phonological recoding pathway more for the prime-phonological target pair than for the prime-non-related target pair. The phonological target, though, is not a straightforward phonological equivalent; it is, in fact, a phonologically reversed form of the target. Therefore, the phonological target might be particularly ambiguous for these participants, bearing both signs of similarity and of difference. Indeed, psycholinguistic research has shown that visually presented codes mandatorily activate a phonological code (e.g., Frost, 1998) and that, moreover, there can be a large variability of visual codes (partially) activating the same phonological code, including e.g., heterographic homophones (soule/sole; e.g., Van Orden, 1987), pseudohomophones (lace/lais; Martin, 1981) but also so-called neighborwords (fruit/flute) and even transposed-letter nonwords (jugde/judge, Perea and Lupker, 2003). There is less research on phonological reversion, though the fact that brain processes for mirror-writing generally remain immature even in adolescents who no longer produce letter reversals (Blackburne et al., 2014) and commonly makes a usually transient return following stroke (e.g., Gottfried et al., 2003), shows the probably easy access to reversed language reading and processing in general. This is confirmed by a study by Saberi and Perrott (1999) who showed that artificially reversing auditory speech segments did not or minimally damage intelligibility.

Hence, high SDS-scorers physiologically react as if they are particularly puzzled by the experimental stimulus and as if they were in some ways double-checking its phonology, i.e., double-checking the ambiguity arisen between e.g., “name” and “main.” This exacerbation of the phonological pathway would then explain the experimental pairs eliciting a more negative N320 than the control pairs. Indication for such a perplexity induced by ambiguity was found before in supraliminal research, as evidence from eye-tracking studies has shown that for homophones the absence of a clue to the contextually appropriate meaning, leads to longer fixation times (Binder and Morris, 1995). For the “non-avoidant” participants, once the target arrives its phonological decoding is facilitated since the phonological code of the prime is still active, and this fact does not seem to be double-checked. As a consequence, there seems to be no big need for them to mobilize the phonologic recoding pathway, and therefore the experimental N320 is smaller (more positive) than the control N320. This hypothesis is corroborated by the finding that the social desirability measure also correlates directly with the N320 amplitude effect: the higher the SDS, the more negative the N320 amplitude effect.

### Language Ambiguity and Defense

The Marlowe-Crowne Social Desirability Scale (Crowne and Marlowe, 1960) was originally intended to be a measure of response bias. Representative items ask participants to respond to typical shortcomings (e.g., “I'm always willing to admit it when I make a mistake”). But the scale was later considered to measure a substantive personality dimension in its own right (Crowne, 1991; Paulhus and Reid, 1991) indexing defensiveness (Crowne and Marlowe, 1964; Weinberger, 1990; Crowne, 1991 p. 18; Paulhus et al., 1998). High scorers are people who deny anything unacceptable about themselves. Weinberger et al. (1979) consider that high SDS scorers resort to defensive inhibition in the face of anxiety when being confronted with unacceptable truths about themselves. In the present study as in others (e.g., Furnham et al., 2002) SDS are interpreted as indexing defensiveness and high vs. low SDS scorers are therefore called high, respectively low defensive participants.

Summarizing our findings, low SDS-scorers or low defensive participants have a more positive N320 upon phonological similarity than upon control and proportionally pick more phonological targets while high defensive participants display a more negative N320 upon phonological similarity than upon control and pick more distracter targets proportionally—and all this is happening unbeknownst to the participant. As the phonological target reveals another way of understanding the prime, maintaining open or opening up its interpretation possibilities, this phonological target elicits ambiguity as concerns the understanding of the prime (and, at the same time, as concerns its proper understanding). Avoiding the phonological target choice unconsciously by the high defensive participants might hence be understood as a way of avoiding ambiguity.

Why would one avoid, or even “defend” against ambiguity? Research from various fields in psychology has shown that ambiguity—defined as “the fact of something having more than one possible meaning and therefore possibly causing confusion” (Cambridge online dictionary)—is more or less aversive depending on personality. For example, the “Intolerance of Uncertainty Scale” (IUS; Freeston et al., 1994) assesses reactions to ambiguous situations and people with high IU are more likely to interpret ambiguous information as threatening (Heydayati et al., 2003) and experience anxiety when engaging in situations with uncertain outcomes (Dugas et al., 2001). A related concept, Tolerance of Ambiguity (TA), measures the tendency to perceive ambiguous situations as sources of threat (for review see Furnham and Marks, 2013). For those with low TA there is an aversive reaction to ambiguous situations including stress, avoidance, delay, suppression, or denial (e.g., Furnham and Ribchester, 1995). Psycholinguistic research in particular, shows that anxious individuals tend to interpret ambiguous information in words or texts in more threatening ways than do non-anxious individuals (e.g., see review in Blanchette et al., 2007).

But apart from being threatening from a content point of view, psycholinguistic research shows ample evidence that phonological ambiguity asks for more mental work as it slows down its processing and makes it less accurate both in visual and auditory paradigms. For a beautiful example, Van Orden (1987) reported that participants made significantly more false positive errors when the homophone mate of the target word was a member of the pre-specified category (e.g., is “rows” a flower?), compared to orthographic controls (is “robs” a flower?). Similar observations are found with other paradigms in heterographic homophones (e.g., Lukatela et al., 1999), with pseudohomophones (e.g., Martin, 1981) and with words with many phonological neighbors (e.g., Gahl and Strand, 2016).

Actually, independently of homophonic effects, language has an inherently ambiguous structure as intermediate or embedded words in sentences are thought to be transiently activated as well (such as the intermediate word “east” and the embedded word “egg” in e.g., “We stop begging,” see Cutler et al., 2002). Therefore, correct decoding of language continually requires an efficient inhibitory mechanism for on-line disambiguation and normal language understanding (e.g., Chiarello, 1985; Burgess and Simpson, 1988; Atchley et al., 1999; Poldrack et al., 1999; Chee et al., 2000). When there is ambiguity, there is an even greater appeal to this energy-consuming inhibitory process (Gernsbacher and Robertson, 1995). Already from this economic point of view, language ambiguity might be considered as aversive. It makes sense to consider that less mental energy is available for allocation to this surplus of mental work, all the more when mental resources are scarce—such as e.g., in case they are needed for defense against socially undesirable judgments. In fact, it has been proposed before that this online inhibitory mechanism that allows for correct disambiguation is a possible neurophysiological correlate of Freudian repression (Bazan, 2012). Both disambiguation from a psycholinguistic point of view (Gernsbacher and Robertson, 1995) and repression from a psychodynamic point of view (Freud, 1915/1957), are considered costly in mental energy. This would fit well with an interpretation of the current results proposing that high SDS-scorers, deemed defensive by us and others, seem indeed physiologically overreactive to ambiguity (i.e., invest a lot in this putative mechanism of repression) while showing behavioral reactions of avoidance (which is the observable defensive counterpart of repression).

### Language Ambiguity and Psychoanalysis

Allowing ourselves for a digression into psychoanalysis at the end of this paper, not only is there more cognitive work required by an ambiguous stimulus but when there is an uncertain interpretation, there is by definition more chance for it to harbor a revealing and potentially threatening interpretation. Listen to how the American physician and poet Oliver Wendell Holmes (1891, p. 11) says it: “People that make puns are like wanton boys that put coppers on the railroad tracks…. They amuse themselves… but their little trick may upset a freight train of conversation for the sake of a battered witticism.” Indeed, confronted with the ambiguous situation of a slip of the tongue—and by extension by any kind of language ambiguity—two reactions are possible: either we acknowledge some recognition of the ambiguous version, and even engage in an interpretation or we might deny any value to the sudden ambiguity and quickly close the ambiguous moment. Listening to potential language ambiguity is, in fact, a working principle for the clinical psychoanalytic work from a Lacanian point of view. To illustrate this principle, we refer to a telling example by the Lacanian analyst Patrick Gauthier-Lafaye (2017, p. 80) who hears an unusual pause in a sentence of a patient. Indeed, in the French sentence: “Ma mère n'était pas parvenue…” (“My mother did not succeed in…”), she pauses in the middle of the word “par-venue” (“succeed”). This slight pause isolates for a suspended moment the embedded phrase “papa revenue,” “daddy has come back.” The analyst does not interpret his patient's sayings but simply repeats “pas par'venue,” opening up a new world of meanings. It appeared that the patient's father left the family without explanation. It had always seemed the minimal duty of the then young woman to be loyal to her mother and to her outrage. Thereby she could never express her own longings for her father to come back, save for this moment in her analysis 40 years later. We have previously proposed an extensive theoretical framework (e.g., Bazan, 2007, 2011, 2012) to understand these psychodynamic logics—the unconscious being structured as a language—in coherence with modern neuroscience and psycholinguitics.

In the present study, we therefore might also propose that high defensive participants shy away from the phonological targets because they potentially harbor threatening ambiguity. What is remarkable is that this pattern is shown to happen unconsciously and totally unbeknownst to the subjects themselves, who are convinced that they make completely arbitrary choices. The results are backed up by the additionally finding that not only do the behavioral responses correlate highly with defensiveness, but also the brain N320 amplitude-effects. High defensive subjects show both different brain and behavior patterns when confronted with phonological ambiguity as compared to low defensive subjects, with a N320 not signaling the phonological closeness between prime and phonological target, but seemingly testifying of some perplexity in decoding the signal. For this reason, we suggest these results are revealing aspects of how personality dispositions, such as defensiveness, interact with elementary mental processes, such as the unconscious resolution of language ambiguity.

### Limitations

A first limitation of the present study is the complexity of the results. First, in the experimental condition, one target is phonologically similar and the other is non-related. We nevertheless take the difference between the N320 reaction upon the experimental trials (one phonologically related and one non-related target) with the N320 reaction upon the control trials (none of the targets is related to the prime), as indicative of its reaction upon the phonological target in the experimental trials specifically. We feel this is justified because it is in fact the only difference between experimental and control trials. Moreover, this complex experimental paradigm allows us to correlate brain waves with behavioral choices in a subliminal paradigm. Second, we do not simply find a N320 reduction indicative of the recognition, and subsequently, the positive choice for the phonological target, we also find a more negative experimental N320, associated with “a negative choice” for (or “avoidance” of) the phonological target. This we interpret as the result of a perplexity in the face of phonological ambiguity, leading to a stronger activation of the phonological recoding pathway.

A second limitation of the study is that indeed no main effects were found. We found a correlation between an ERP and a behavioral effect, but we did not find a significant difference for the whole group between experimental N320 and control N320. In some ways, the present results are similar to the Snodgrass et al. (1993) study: there seems to be, in the total pool of participants, two groups with opposite behavior: one group in which the recognition of the phonological similarity leads to lesser mobilization of the phonological recoding pathway and therefore a relative N320 reduction; these participants tend to pick the phonological targets; and another group in which the detection of the phonological ambiguity leads to over-mobilization of the phonological recoding pathway and therefore a relative N320 increase; these participants tend to avoid the phonological target. These bidirectional effects, then, neutralize the main effects.

## Conclusion

In conclusion, the amplitude of a subliminally induced mid-left brain N320 wave, known to react upon supraliminal phonological mismatch, relative to its behavior in a control condition, significantly predicts the behavioral choices of the participant more than half a second later, while the subjective experience of the participants is one of complete arbitrariness. Moreover, social desirability, as measured by Crowne and Marlowe's Personal Reaction Inventory, significantly correlates with both the behavioral and the N320 brain responses of the participants. It is proposed that in participants with low social desirability scores the phonological target induces a normal N320 reduction that increases their probability to pick this target, while participants with high social desirability scores have a perplexed brain reaction upon the phonological target with a negatively peaking N320 that more often leads them to avoid this target. Social desirability, which is understood as reflecting defensiveness, might also manifest itself as a defense against the (energy-consuming) ambiguity of language. The specificity of this study is that all of this is happening totally out of awareness and at the level of very elementary linguistic distinctions.

## Ethics Statement

This study was carried out in accordance with the recommendations of the Institutional Review Board of the University of Michigan (Department of Psychiatry) with written informed consent from all subjects. All subjects gave written informed consent in accordance with the Declaration of Helsinki. The protocol was approved by the Institutional Review Board.

## Author Contributions

AB had the idea for the research, did the experiments, the data-analysis, and the write-up. RK did the ERP measurements and ERP data analyses. EW helped with the preparation and the administration of the experiments. JS taught and supervised the subliminal priming method and supervised the data analyses. LB helped with the rationale of the research and the interpretation of the results. HS supervised the whole process at all stages. All authors discussed preparation, running, data-analyses and interpretation of the study during the Shevlab-meetings.

### Conflict of Interest Statement

The authors declare that the research was conducted in the absence of any commercial or financial relationships that could be construed as a potential conflict of interest.

## Footnotes

^1^For example, Freud (1900/1958: 560) qualifies the homophony dysenteria/diphteria in one of his dreams (≪ Irma's injection ≫) as a ≪ paraphasic assonance ≫.

^2^This example is given by Dr. A. A. Brill.

^3^Our translation of: ≪ On se croit en général libre de choisir les mots et les images pour exprimer ses idées. Mais une observation plus attentive montre que ce sont souvent des considérations étrangères aux idées qui décident de ce choix et que la forme dans laquelle nous coulons nos idées révèle souvent un sens plus profond, dont nous ne nous rendons pas compte nous-mêmes. (…) certaines de ces images et manières de parler sont souvent des allusions à des sujets qui, tout en restant à l'arrière-plan, exercent une influence puissante sur celui qui parle. Je connais quelqu'un qui, à une certaine époque, se servait à chaque instant, (…) de l'expression suivante: ‘Lorsque quelque chose traverse tout à coup la tète de quelqu'un.' Or, je savais que celui qui parlait ainsi avait reçu, peu de temps auparavant, la nouvelle qu'un projectile russe avait traversé d'avant en arrière le bonnet de campagne que son fils, soldat combattant, avait sur la tête. ≫.
