# Supplementary material for: Phonological Ambiguity Detection Outside of Consciousness and Its Defensive Avoidance
Source: Front Hum Neurosci. 2019 Apr 5;13:77. doi: 10.3389/fnhum.2019.00077 (PMC6460346; doi:10.3389/fnhum.2019.00077)
Supplement: Supplementary file 1 [file Data_Sheet_1.docx]

Supplementary Materials

**Table S1**. **Lists of phonological targets and distracters.** Lists of primes (and phonological transcriptions) with their phonological target (exact phonological reverses of the primes), respectively their non-related target alternatives; OS: Weber’s orthographic similarity index (Weber. 1970) between prime and phonological target (OS_phon_) and between prime and distracter (OS_dis_)^1^.

LIST 1

|  | **prime** | **phon** | **dis** | **OS_phon_** | **OS_dis_** |
| --- | --- | --- | --- | --- | --- |
|  | BACKS/baks | SCAB/skab | HOUSE | 262.22 | 70.00 |
|  | BOSS/bŏs | SOB/sŏb | MATH | 294.64 | 50.00 |
|  | CAME/kām | MAKE/māk | DUST | 380.00 | 50.00 |
|  | CAT/kat | TACK/tak | LOUD | 294.64 | 37.50 |
|  | CHANCE/chăns | SNATCH/snăch | HORSE | 250.00 | 258.03 |
|  | COTS/kŏts | STOCK/stŏk | HARM | 328.89 | 50.00 |
|  | FINE/fīn | KNIFE/nīf | DOUBT | 442.22 | 40.00 |
|  | ICE/īs | SIGH/sī | LOCK | 66.07 | 66.07 |
|  | LIED/līd | DIAL/dīl | EARTH | 125.00 | 62.22 |
|  | LOOKS/lüks | SCHOOL/skül | PLANT | 314.39 | 70.00 |
|  | LUCK/lŭk | CULL/kŭl | PAST | 275.00 | 50.00 |
|  | MAD/mad | DAMN/dam | EASE | 294.64 | 66.07 |
|  | MAID/mād | DAME/dām | REST | 200.00 | 50.00 |
|  | MOAN/mōn | GNOME/nōm | CUPS | 173.33 | 50.00 |
|  | MOLE/mōl | LOAM/lōm | GRID | 200.00 | 50.00 |
|  | MUD/mŭd | DUMB/dŭm | FAKE | 294.64 | 37.50 |
|  | NICK/nik | KIN/kin | ART | 208.93 | 37.50 |
|  | OAF/ōf | FOE/fō | RUN | 116.67 | 50.00 |
|  | ROPE/rōp | POUR/pōr | LIST | 200.00 | 50.00 |
|  | SAKE/sāk | CASE/kās | ITCH | 380.00 | 50.00 |
|  | SAME/sām | MACE/mās | FEET | 380.00 | 75.00 |
|  | SELL/sel | LESS/les | ROOM | 275.00 | 50.00 |
|  | SHALL/shăl | LASH/lăsh | MONK | 306.67 | 40.00 |
|  | SOON/sün | NOOSE/nüs | BLACK | 440.00 | 40.00 |
|  | SPILL/spĭl | LIPS/lĭps | BOAT | 328.89 | 40.00 |
|  | SPITE/spīt | TYPES/tīps | DREAM | 130.00 | 70.00 |
|  | SUCK/sŭk | CUSS/kŭs | WAKE | 275.00 | 75.00 |
|  | TILL/til | LIT/lit | YELL | 294.64 | 480.00 |
|  | TOAD/tōd | DOTE/dōt | PULP | 200.00 | 50.00 |
|  | VAIN/vān | KNAVE/nāv | STRIP | 173.33 | 62.22 |

LIST 2

|  | **prime** | **phon** | **dis** | **OS_phon_** | **OS_dis_** |
| --- | --- | --- | --- | --- | --- |
|  | CUTS/kŭts | STUCK/stŭk | MOP | 170.00 | 37.50 |
|  | CAUGHT/kôt | TALK/tôk | MIND | 73.33 | 33.33 |
|  | CLUB/klŭb | BULK/bŭlk | MOON | 275.00 | 50.00 |
|  | DOLE/dōl | LOAD/lōd | CHEW | 200.00 | 75.00 |
|  | DOUGH/dō | ODE/ōd | LINK | 155.00 | 40.00 |
|  | KNIT/nit | TIN/tin | BAR | 294.64 | 37.50 |
|  | KNOCK/näk | CON/kän | GAS | 255.00 | 30.00 |
|  | LAKES/lāks | SCALE/skāl | PUNCH | 190.00 | 50.00 |
|  | LICKS/lĭks | SKILL/skĭl | CHEAP | 250.00 | 70.00 |
|  | LOBE/lōb | BOWL/bōl | HUGE | 200.00 | 255.00 |
|  | LORE/lōr | ROLL/rōl | HAIR | 275.00 | 75.00 |
|  | MAIL/māl | LAME/lām | CROSS | 200.00 | 40.00 |
|  | MORE/mōr | ROAM/rōm | HELP | 200.00 | 75.00 |
|  | NAME/nām | MAIN/mān | SOFT | 200.00 | 50.00 |
|  | NEAT/nēt | TEEN/tēn | GLASS | 200.00 | 62.22 |
|  | NICE/nīs | SIGN/sīn | BELT | 100.00 | 75.00 |
|  | OWN/ōn | NO/nō | SIT | 110.00 | 50.00 |
|  | PATCH/păch | CHAP/chăp | KEEN | 306.67 | 40.00 |
|  | PICKS/pĭks | SKIP/skĭp | CHAIR | 262.22 | 90.00 |
|  | PITCH/pich | CHIP/chip | LUNG | 306.67 | 40.00 |
|  | ROOT/růt | TOUR/tůr | BLANK | 200.00 | 40.00 |
|  | SAP/săp | PASS/păs | FUND | 294.64 | 37.50 |
|  | SEEP/sēp | PEACE/pēs | BLIND | 173.33 | 40.00 |
|  | SIDE/sīd | DICE/dīs | TANK | 380.00 | 50.00 |
|  | SKAT/skăt | TAKS/tăks | DEEP | 180.36 | 50.00 |
|  | SPOKE/spōk | COPES/kōps | MEAL | 190.00 | 62.22 |
|  | TAN/tăn | GNAT/năt | YIELD | 294.64 | 30.00 |
|  | TELL/tel | LET/let | JAM | 294.64 | 37.50 |
|  | TOOL/tül | LUTE/lüt | BATH | 100.00 | 75.00 |
|  | ZOOM/züm | MOOS/müz | CHAIN | 400.00 | 40.00 |

^1For four targets out of 60 (5. 8. 28 and 40) it appeared that the orthographic similarity of the distracter item with the prime was. in fact. equal or higher than was the orthographic similarity of the phonological target with the prime.^

**Figure S1**. **No influence of stimulus detectability on participant’s behaviour**. Net number of phonological choices (experimental – control) in function of the d’ detectability parameter (r=.05; p=.783; *N* = 31) showing there is no relationship between the experimental behavioral effect (net number of phonological choices) and the detectability of the stimuli (prime and targets). This, together with the d’ not different from zero (.044 ± .047), confirms that participants made their choices below the threshold for conscious perceptions at stringent conditions of subliminality.


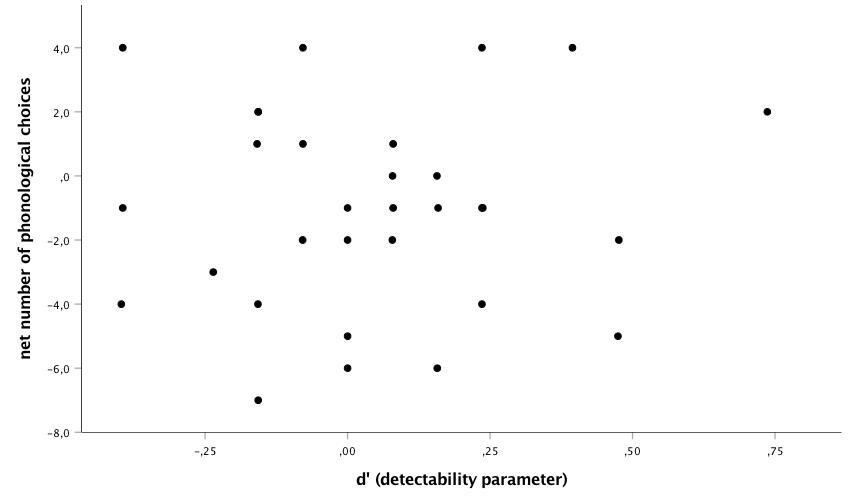


**Table S2**. **No main effect between Experimental and control N320 amplitudes**. Amplitudes in μV (Mean ± Standard Error of the Means) in experimental versus control conditions at all electrodes; p-values show that there are no main brain effects when experimental trials (with phonological similarity) are contrasted with control trials (without similarity); *N* = 31.

| electrode | experimental | control | *p-value* |
| --- | --- | --- | --- |
| F_P1_ | -.37 ± .17 | -.31 ± .16 | .724 |
| F_P2_ | -.32 ±. 16 | -.28 ± .15 | .773 |
| F_3_ | -.61 ± .20 | -.64 ± .18 | .837 |
| F_Z_ | -.69 ±.21 | -.64 ± .22 | .788 |
| F_4_ | -.58 ± .20 | -.49 ± .19 | .641 |
| C_Z_ | -.66 ± .21 | -.74 ± .20 | .747 |
| T_3_ | -.46 ± .13 | -.48 ± .12 | .879 |
| T_4_ | -.25 ± .12 | -.22 ± .09 | .831 |
| T_5_ | -.39 ± .14 | -.33 ± .13 | .691 |
| T_6_ | -.29 ± .13 | -.25 ± .11 | .843 |
| P_Z_ | -.64 ± .21 | -.54 ± .17 | .689 |
| P_3_ | -.54 ± .19 | -.49 ± .16 | .830 |
| P_4_ | -.48 ± .19 | -.38 ± .14 | .624 |

**Figure S2. The higher the defensiveness, the more negative the N320 the brain effect.** N320 amplitude effect (experimental - control) at the mid-left of the brain (average of F_P1_, F_3_, F_Z_, C_Z_, T_3_, T_5_, P_Z_ and P_3_) in function of Marlowe Crowne’s Social Desirability Scores; r = -.49; p = .005; *N* = 31.

**
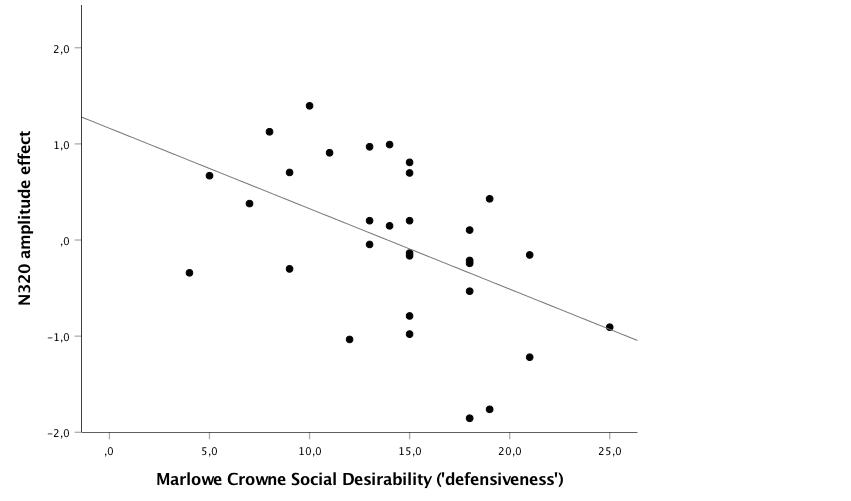
**

**Table S3**. **No correlations between N320 amplitude and orthographic similarity.** Pearson’s correlations between the N320 amplitude and OS_phon_ (orthographic similarity between prime and phonological target); *N=*60.

|  | F_P1_ | F_P2_ | F_3_ | F_Z_ | F_4_ | C_Z_ | T_3_ | T_4_ | T_5_ | T_6_ | P_Z_ | P_3_ | P_4_ |
| --- | --- | --- | --- | --- | --- | --- | --- | --- | --- | --- | --- | --- | --- |
| r | **-.24** | **-.22** | **-.23** | -.18 | -.18 | -.09 | -.11 | -.01 | -.05 | -.08 | -.04 | -.03 | -.01 |
| *p value* | ***.061*** | ***.086*** | ***.074*** | *.172* | *.168* | *.512* | *.385* | *.932* | *.688* | *.550* | *.780* | *.813* | *.971* |
